# Central Nervous System Reservoirs and HIV Cure: Therapeutic Opportunities and Challenges

**DOI:** 10.1007/s11904-026-00798-9

**Published:** 2026-10-17

**Authors:** Thomas A. Angelovich, Michael Roche, Paul R. Gorry, Melissa J. Churchill

**Affiliations:** 1 ATRACT Research Centre, School of Health and Biomedical Sciences, Royal Melbourne Institute of Technology (RMIT) University, Bundoora West Campus, 225-245 Plenty Rd, Bundoora, Melbourne, VIC 3083 Australia; 2 Life Sciences, Burnet Institute, Melbourne, VIC Australia; 3 Department of Infectious Diseases, The University of Melbourne at The Peter Doherty Institute for Infection and Immunity, Melbourne, VIC Australia

**Keywords:** Central nervous system, Reservoirs, Cure, Latency, Neuroinflammation

## Abstract

**Purpose of review:**

Despite viral suppression with antiretroviral therapy, people with HIV (PWH) remain at increased risk of neurocognitive impairment, neuroinflammation, and potential viral rebound following therapy interruption. Although the central nervous system (CNS) is a recognised HIV reservoir, it is often not fully considered in cure approaches.

**Recent findings:**

HIV DNA and ongoing viral transcription persist in the CNS of ART-suppressed PWH, potentially contributing to neuroinflammation and neurocognitive dysfunction. HIV cure strategies, including latency-reversing/promoting agents, broadly neutralising antibodies and other approaches have varying degrees of CNS penetration, efficacy and potential neurotoxicity. Therefore, interventions aimed at silencing or eradicating HIV reservoirs require monitoring of both reservoir-targeting efficacy and neurological adverse effects.

**Summary:**

Greater consideration of CNS penetration, efficacy in targeting CNS reservoirs, and neurotoxicity is required in HIV cure studies. Developing CNS-inclusive strategies that minimise adverse neurological effects may be an important step in achieving HIV cure.

## Introduction

Effective viral suppression with antiretroviral therapy (ART) has transformed HIV into a more manageable chronic infection in well-resourced environments. However, treatment is not a cure and fails to eliminate persistent virus meaning the ART-suppression is required for life [1]. Further, people with HIV (PWH) have elevated risk and incidence of HIV-associated comorbidities including cardiovascular disease (CVD), bone and metabolic disease, renal disease, malignancies and importantly, neurocognitive disease [2–4]. HIV infection of the central nervous system (CNS) remains a major challenge for cure research mainly because it is difficult to sample, contains diverse reservoir cell types, and is separated from the periphery by the blood brain barrier (BBB), that limits immune surveillance and therapeutic delivery [5–8]. Despite these challenges, it is important that HIV cure strategies evaluate more than reductions in peripheral measures of HIV persistence [9] and instead, aim at reducing residual viral activity and inflammation throughout the body including the brain in order to avoid treatment-induced neuronal harm, and preserve or improve neurological health.

## Considerations for Directly Targeting HIV Persistence in the CNS

### The CNS is a Unique and Heterogeneous HIV Reservoir

The CNS remains a heterogeneous site of HIV persistence despite viral suppression in the blood with ART [10–13]. A recent review integrating data from human autopsy studies conducted using biospecimens obtained from the National NeuroHIV Tissue Consortium (NNTC) and Last Gift cohort estimated the HIV DNA reservoir in parenchymal brain tissue of ART-suppressed PWH as 2.53 and 4.08 copies/10^6^ cells, respectively [14]. As observed in peripheral reservoirs, considerable inter-individual variability exists in reservoir size, likely reflecting factors including length of infection, time to ART initiation and also viral strain including co-receptor tropism, although this remains incompletely understood. HIV persists in long-lived cell populations in the brain including mainly in resident microglia and perivascular macrophages, alongside lower-level persistence in astrocytes and pericytes [6, 7, 15–18]. HIV persistence may also involve infiltrating HIV-infected CD4 + T cells passaging from the circulation, especially in individuals with a compromised BBB [19]. Fundamental differences in these cells likely contribute to their role in persistence. CNS cell populations differ in lifespan, anatomical localization, transcriptional regulation, inflammatory responsiveness, and susceptibility to immune-mediated clearance. This heterogeneity of cell types as well as unique HIV phenotypes, have direct implications for cure design. For example, strategies that are effective against latently infected CD4 T cells may have limited impact on infected myeloid cells including brain-resident microglia and perivascular macrophages [20, 21]. Furthermore, an intervention that induces HIV transcription, translation and immune-mediated clearance in the blood, may inadvertently increase viral RNA and/or protein expression without efficiently eliminating the infected cell in the brain. Eradication and cure strategies must achieve high cellular precision to target these distinct myeloid populations without triggering toxic, off-target effects in neighbouring, non-infected neurons. It follows that cure approaches must therefore account for both the infected cell type and the local neuroimmune environment in which that cell persists.

### Relevance of Defective HIV Proviruses

Defective HIV proviruses are potentially highly relevant contributors to ongoing neuropathology in the brain. Several studies have clearly demonstrated that similar to the T cell reservoir, most integrated HIV genomes detected in the CNS are non-intact and likely replication defective [10, 12, 15, 22, 23]. Importantly, a subset of defective HIV genomes do retain transcriptional activity and may also generate HIV proteins, which results in the activation of antiviral and inflammatory pathways despite a loss of replication competence [22, 24–28]. In the CNS, where even low-level glial activation or exposure to viral products may alter synaptic and neuronal health, a HIV provirus need not be rebound competent to remain clinically relevant.

### Anatomical Distribution

HIV distribution is highly heterogeneous across anatomical brain regions and cerebrospinal fluid (CSF) spaces. Postmortem human tissue data demonstrates that latent viral seeding and viral DNA loads vary significantly between areas like the frontal cortex, basal ganglia, and occipital cortex [10, 13, 14]. Therapeutic interventions must therefore achieve widespread, uniform penetration to ensure no regional tissue pockets or immune-privileged sanctuaries evade clearance.

### Brain Penetration

The brain HIV reservoir is anatomically and phenotypically diverse. Therapeutic agents must successfully cross the BBB and choroid plexus to achieve therapeutic concentrations in complex brain tissues. Most traditional small-molecule drugs, broadly neutralizing antibodies (bNAbs), and engineered genetic vectors used in gene editing have poor bioavailability in the brain compartment with significantly lower levels in CSF and parenchymal tissue relative to plasma [29–32]. There is significant work being done to address the issue of delivery including lipid nanoparticles, cell-penetrating peptides, brain-shuttle biologics and optimized adeno-associated viral vectors (like AAV9) [33–39], but further insight is required in the context of HIV.

### Neurological Safety

The CNS is an immunologically delicate site where standard viral clearance mechanisms can exacerbate HIV-associated neuroinflammation and may cause irreversible damage. Forcing viral reactivation through “shock and kill” followed by analytical treatment interruption approaches with latency-reversing agents including disulfiram and vorinostat risks releasing neurotoxic viral proteins (such as Tat and gp120), which can potentially accentuate/accelerate neurocognitive disorders and injury [40–45]. Studies using JAK/STAT inhibitors including baricitinib and ruxolitinib (discussed further below) have shown promise with demonstrated efficacy in vitro and in humanized animal models with limited evidence of neurotoxicity in PWH [46, 47]. Notably, baricitinib is currently undergoing clinical assessment for impact on the CNS reservoir in PWH with the resulting data likely to provide important insights into tolerability and efficacy in vivo [48]. Consequently, safe curative approaches must maintain a strict balance between effective HIV reservoir reduction and the prevention of permanent cognitive or structural brain injury, making alternative strategies like epigenetic silencing (“block and lock”) highly attractive [49].

## Strategies that Directly Target HIV

### Reactivate and Eliminate

Shock-and-kill approaches to HIV cure involve the reactivation of latent HIV expression and subsequent removal of the activated infected cells either by viral cytopathic effects or immune-mediated clearance [50]. In theory, this could activate otherwise silent proviruses in CNS-resident cells, specifically myeloid populations, which likely will not behave like peripheral T-cell reservoirs. Brain microglia are persistently infected with replication-competent macrophage tropic HIV, as well as defective HIV, during prolonged ART suppression [7, 15]. The latency of HIV in microglia has been demonstrated to be regulated by epigenetic pathways including CoREST/HDAC repression and TLR3/IRF3 innate-immune signalling, that are functionally distinct from those governing CD4 + T-cell reservoirs [49, 51, 52]. Recently, treatment of latently infected cells CD4 + T cells or microglia isolated from PWH or SIV-infected NHPs with the selective histone decrotonylation inhibitor citarinostat induced HIV transcription [53], demonstrating a promising alternate pathway to viral reactivation in brain cells.

Activation of HIV in microglia initiates or enhances a pathological loop driven by cellular crosstalk which may damage neighbouring neurons, causing a measurable structural loss of dendrites [54]. Crucially, neuronal injury may perpetuate further damage as dysfunctional neurons release molecular distress signals while losing their ability to emit normal inhibitory cues, and this can cause continued activation of adjacent microglia. Additionally, activation of HIV within the CNS environment causes astrocyte dysfunction resulting in impaired glutamate uptake and predisposing the peri-synaptic environment to excitotoxicity [55]. It has also been demonstrated that certain latency reversal agents (LRAs) alone can elicit proinflammatory cytokine secretion as well as complement upregulation in astrocytes, independent of direct viral activity [40]. It follows that reactivation of latent HIV within the CNS in the absence of an efficient CNS-targeted ‘kill’ mechanism is unlikely to reduce the CNS reservoir and may result in CNS harm.

### Long Term Silencing

Block-and-lock strategies are focussed on long term transcriptional repression of HIV rather than induced viral reactivation using latency-promoting agents (LPAs). This strategy is particularly attractive in the brain as reducing viral RNA and protein production would not only decrease rebound potential and but also impact the chronic inflammation due to residual viral expression. Using BRD4-selective modulators and the deactivated CRISPR system, dCas9–KRAB, viral transcription from integrated HIV genomes can be inhibited, at least in microglial and myeloid cell models [56, 57]. Suppression of transcription using the Tat inhibitor didehydro-cortistatin A has been demonstrated in latently-infected CD4 + T cells from ART-suppressed PWH [58, 59] and importantly shown to suppress viral rebound in ART-suppressed HIV-infected humanized mouse models including suppression of viral RNA in the brain [60]. Inhibitors of RNA Pol II mediator kinases CDK8 and CDK19 impair recruitment to the HIV LTR [61], therefore, suppressing viral transcription, although this has not been verified in brain cells.

The ability to repress transcription from HIV genomes may be even more important in the case of defective HIV proviruses. For example, intron-containing HIV RNA produced from defective HIV genomes can drive interferon signalling pathways in microglia and myeloid cells in the absence of intact replication-competent genomes [28, 62]. Given that viral transcripts and proteins also persist in brain cells of ART-suppressed PWH [63], durable suppression of viral products rather than physical provirus removal could be desirable as a therapeutic as well as a functional cure strategy. One important consideration, however, is whether this type of silencing can be maintained in long-lived brain cells especially under conditions where inflammatory signals have been consistently demonstrated to reactivate microglial proviral transcription [64]. Moreover, ageing further impacts epigenetic stability which may undermine the efficacy of treatment long-term [65].

### Proviral Inactivation or Removal

Gene-editing and proviral genome inactivation strategies purport to offer a more definitive intervention through targeted excision, disruption of essential viral sequences, or a permanent block to proviral expression. Systemic delivery of AAV9-CRISPR/Cas9 in ART-treated simian immunodeficiency virus (SIV)-infected macaques produced targeted proviral excision in multiple tissues including brain, albeit with reduced entry relative to peripheral tissues [39, 66, 67]. CRISPR editing targeting both CCR5 and the HIV-1 L-Gag regions have also eliminated detectable proviral DNA in CNS tissues in 58% of treated humanised mice [68]. Despite these promising findings, the CNS still poses stringent requirements for delivery and safety as a recent study has detected the generation of a neuroepithelial tumour in a 5 year old child, 4 years follow AAV therapy highlighting the need for constant review [69].

Any editing strategy also needs to reach the relevant cell types and anatomical sites at sufficient levels while minimising off-target effects, inflammatory responses, and potential toxicity. Studies using engineered AAV capsid demonstrated variation in myeloid cell transduction across brain regions, suggesting that standard systemic vectors may not access microglial or perivascular macrophage reservoirs [70]. In addition to potential safety issues [69], incomplete distribution could therefore leave anatomically isolated or myeloid-associated reservoirs untouched, limiting the effect of otherwise promising interventions. Therefore, while evidence in animal models provides proof-of-concept of removal of HIV proviral DNA in the brain, significant hurdles regarding safety, delivery, efficacy and regulatory approval must be overcome before these approaches can be translated into clinical practice, making implementation unlikely in the short-term.

### Immune-Mediated Clearance

In the peripheral compartment, bNAbs, therapeutic vaccines, engineered cellular therapies, and innate immune-based therapies are being used to enhance recognition, targeting and facilitate elimination of HIV-infected cells [71–74]. bNAbs can trigger antibody-dependent cellular cytotoxicity (ADCC) of infected targets through FcγRI-positive monocytic effectors, and Fc-engineering modifications, including afucosylation and GASDALIE mutations, further augment ADCC and antibody dependent cellular phagocytosis (ADCP) potency against infected cells [74–78]. These strategies are likely to be most useful in combination with latency reversal or other methods that increase antigen visibility.

However, immune clearance in the CNS raises additional problems in the CNS. Antibody and effector-cell access to the CNS is constrained by the BBB, infected cells in the CNS generally express little antigen, and inflammatory killing could damage surrounding neural tissue, specifically the potential for immune effectors entering the brain to enhance encephalitis before viral clearance is complete [29–31, 79, 80]. Resident myeloid reservoirs likely require recognition and elimination mechanisms that differ from those optimised for infected CD4 T cells. Engineered cellular therapies such as CAR-T cells can reach and activate CNS myeloid compartments, as demonstrated by IFNγ-dependent myeloid remodelling in intracranial tumour models [81], however, this likely constitutes a mechanism of enhanced inflammation.

## Strategies that Do Not Directly Target HIV

Not all cure strategies are aimed at directly eliminating infected cells or HIV proviral DNA. In the CNS, interventions that reshape the surrounding neuroimmune environment may reduce the neuroinflammation caused by viral persistence or contributed by direct therapies and may also alter inflammatory conditions that support intermittent HIV transcription and reservoir maintenance.

### Modulating Neuroinflammation

Even under effective ART, persistent microglial activation due to residual HIV transcription drives neuronal dysfunction. In SIV-infected macaques, upregulation of CSF1R in activated microglia correlated with oxidative stress and CNS pathology, suggesting that myeloid activation sustains neuroinflammation independently of viral replication [82]. Subsequent studies treating SIV-infected rhesus macaques with the CSF1R kinase inhibitor BLZ945 demonstrated significant decreases in perivascular macrophages and reduce levels of viral DNA in the brain [83]. However, it is of note that levels of microglia were not impacted. HIV RNA and Tat protein further impact this state. Viral RNA engages microglial TLR7/8, recruiting IRAK1 to drive TRAF6/TAK1-dependent cytokine and chemokine release, while Tat enhances P2Y4 receptor signalling in astrocytes, triggering NF-κB- and ERK-dependent IL-6 production and hippocampal neuronal apoptosis [84, 85]. Notably, studies testing the JAK 1/2 inhibitor Baricitinib (currently under Phase IIb clinical trial NCT05452564) demonstrated a reduction of infected myeloid cells in the CNS and improved neurocognitive outcomes in murine models [46].

Innate immune activation may create a self-perpetuating inflammatory response. TLR3 stimulation in microglia reactivates latent HIV via IRF3 recruitment to the LTR rather than classical NF-κB activation, linking neuroinflammation to proviral transcription [51]. Disrupting this axis using IRAK1 inhibitors or Nurr1 agonists recruits repressive complexes to induce epigenetic proviral silencing and suppress inflammatory genes, highlighting the need for therapies that curb pathological activation while preserving neuroprotective functions [49, 84]. Achieving immune environment homeostasis in the CNS reservoir requires strategies that simultaneously restrain pathology causing activation and maintain physiological neuroprotective microglial functions.

### Restoring Barrier Function and Therapeutic Access

BBB integrity is compromised during HIV infection by HIV gp120 and Tat which disrupt tight junction proteins (ZO-1, occludin, and claudin-5) in a mechanism that involves CCR5-mediated PKC activation and oxidative MMP-driven degradation, resulting in an increase in permeability and monocyte transmigration into the CNS [86, 87]. Soluble CD40L further downregulates claudin-5, implicating peripheral immune signals as drivers of CNS entry and secondary inflammatory injury [88].

Restoring BBB integrity while improving therapeutic access appears contradictory but are distinct, complementary priorities. PPARα and PPARγ agonists attenuate tight junction loss by inhibiting endothelial MMP and proteasome pathways, demonstrating that barrier repair is feasible [87]. For improved CNS drug delivery, ApoE receptor-targeted nano-NRTIs enhanced brain antiviral activity with low neurotoxicity in experimental infection models, demonstrating that ligand-directed nanoplatforms can cross the BBB for effective therapeutic delivery [89]. Restoring BBB function while allowing for therapeutic access are likely mechanistically separate goals but essential during cure strategy development.

### Neuroprotection and Brain Circuit Repair

Infected myeloid cells and HIV proteins drive neuronal injury through overlapping mechanisms. Tat and secreted gp120 cause neuronal DRP1-dependent mitochondrial fragmentation and ROS accumulation, leading to neuronal dendrite degeneration, while HIV-infected macrophages facilitate oxidative stress which leads to mitochondrial release of excess glutamate and excitotoxic neuronal death [90, 91]. Given these converging injury pathways, it is important that neuronal and synaptic measures of CNS health be core goals of any cure intervention that impacts myeloid or glial biology. Experimental interventions that target these pathways have demonstrated stand-alone neuroprotection and combined anti-viral/neuroprotection. Dimethyl fumarate inhibits HIV replication in macrophages and enhances the HO-1 antioxidant response, diminishing macrophage neurotoxicity in vitro [92]. Nanoparticles that accumulate in the brain and deliver antiretrovirals and antioxidant agents can reduce CNS HIV burden, astrocyte/microglial oxidative stress and neuroinflammation in vivo in an EcoHIV mouse model, showing that combined approaches can reasonably expand the safety window for direct reservoir targeting interventions [93].

### Reducing Systemic Contributors to CNS Vulnerability

CNS risk in PWH is shaped not only by local viral persistence but by interacting systemic drivers including vascular disease, metabolic dysfunction, ageing, and chronic immune activation. For instance, subclinical carotid intima-media thickness serves as an independent predictor of impaired psychomotor speed and memory function in ART-suppressed men [94]. This relationship highlights a dual-pathway mechanism, demonstrating that HIV-driven chronic inflammation and metabolic changes cause progressive vascular injury that occurs concurrently with direct viral insults to the CNS. Peripheral immune activation and its impact on the CNS begins early. HIV RNA is detectable in CSF within days of exposure [95], and active viral transcription in CSF CD4 + T cells during acute infection correlates with local inflammatory signalling [96]. Early and sustained ART remains critical. Initiation during acute infection normalises CSF neopterin, CXCL10, and CCL2 by 24 weeks [97, 98] and reduces CSF anti-HIV antibody titres approximately 43-fold compared with chronically treated infection [98], indicating a meaningful dampening of CNS inflammatory burden if treatment begins before reservoir seeding is fully established, reducing size and cellular diversity. It is important to also recognise that systemic inflammation and injury also may contribute to neuroinflammation, even in the absence of HIV. Notably, we demonstrated in non-human primates that gut dysfunction, a hallmark of early HIV infection, is associated with neuroinflammation [99], supporting the impact of systemic processes on neuropathology.

Together, this evidence contextualises rather than diminishes HIV cure-directed approaches. Long-term brain health reflects cumulative and interacting insults, and strategies that attenuate vascular injury, metabolic dysregulation, or systemic immune activation may reduce the biological environment that sustains CNS viral persistence and amplifies neuronal injury.

## Combination Strategies are Likely to be Necessary

The most plausible route to a CNS-inclusive cure is a rational combination approach rather than reliance on a single intervention. The core question is not only which therapy targets HIV, but which sequence of interventions is necessary to preserve neurological safety. A staged model may be required. This may involve stabilizing the neuroimmune environment and optimize delivery first, then suppressing, exposing or disabling persistent virus, and finally enable controlled clearance whilst monitoring for CNS inflammation and neuronal stress. It may be safer to activate this sequence than to introduce a powerful reservoir-perturbing intervention into an already compromised CNS.

## Preclinical Models Must Become More CNS Relevant

Advances in HIV cure strategies necessitate model systems that can appropriately assess relevant nuances in brain reservoir biology. Although peripheral latency models are critical for validating interventions on peripheral reservoirs and toxicity, they cannot adequately recreate myeloid cells that reside in the brain, BBB biology, glial-neuronal crosstalk, and low-level viral expression on neural health. Current human models of the brain, and more specifically, HIV infection of the brain use cellular systems to facilitate mechanistic studies, and include induced pluripotent stem cell-derived (iPSC) microglia, astrocytes, neurons can be used to generate multicellular assembloids and organoids [100–102]. These, however, can be limited by the cellular composition, architecture and altered growth dynamics and in some cases longevity [103], although a recent study demonstrated long-term viability and useability of human brain organoids consisting of cortical neurons out to 5 years in culture with evidence of maturation with time [104]. Ex vivo tissue systems including organotypic brain models further extend the 3D architecture and cellular composition of the brain. An adult human brain tissue culture system has been reported and demonstrated to allow for ex vivo infection in the presence or absence of ART [105]. Animal models including non-human primate models with SIV or simian-human immunodeficiency virus (SHIV) have also provided a wealth of specific data on distribution of the reservoir, pharmacology and neuroimmune responses in a more controlled manner than those ex vivo [7, 99, 106, 107]. However, limitations exist with all systems, meaning no one-fits-all approach is currently available.

Importantly, understanding the virus that constitutes both the blood, and the CNS reservoir is essential [11]. The long-terminal repeat (LTR) plays a central role in many proposed cure strategies discussed. Understanding similarities and/or differences in genomic sequence, inducibility, responsiveness to inflammatory cues, and sensitivity to silencing strategies is essential [19, 23, 108, 109]. Such studies may also help separate rebound-relevant genomes from defective genomes that remain transcriptionally active and biologically important. Fully understanding the relationship between the peripheral reservoir and the brain is also an important component of delivering safe cure strategies. The ability of peripheral HIV activity to impact neuroinflammation has been demonstrated in a number of studies. It follows that cure strategies targeting peripheral reservoirs will also impact the CNS.

## Endpoints for CNS-Inclusive Cure Studies

While peripheral measures of HIV reservoir size, activity and inflammation remain essential in HIV cure studies, they are not generally sufficient to establish CNS efficacy or safety. Monitoring the efficacy of HIV cure studies on the CNS reservoir remains challenging as the CNS is difficult sample directly, and no gold-standard biomarker of CNS infection, reservoir activity, or neuroinflammation exists. Despite these limitations, neuroimaging techniques and cognitive panels can provide important clinical evidence of impact and potential neurotoxicity in PWH. Measures of CSF reservoir size, viral transcripts and neuroinflammation can also be measured to provide surrogate measures of the CNS. Therefore, cure studies should incorporate CNS-relevant endpoints from the earliest stages of development to evaluate the efficacy and safety in the CNS. Useful endpoints may include:

- *Virological endpoints*: cell-associated HIV DNA and RNA in CSF
- *Safety endpoints*: neurological symptoms, neuropsychiatric effects, CSF inflammation, treatment-associated immune activation, and evidence of neuronal injury.
- *Neurobiological endpoints*: markers of neuronal and glial injury, imaging measures, synaptic or metabolic indicators, and longitudinal cognitive assessment.
- *Functional cure endpoints*: rebound during carefully monitored treatment interruption, interpreted alongside CNS outcomes rather than plasma virology alone

A reduction in systemic reservoir markers without improved CNS outcomes may not represent meaningful progress for the brain. Notably, an intervention that suppresses viral transcription, reduces neuroinflammation, and preserves brain health may be therapeutically valuable even if it does not remove every integrated provirus.

## Priorities for the Field

Several key priorities exist for the field which include:

- Define which CNS-associated infected cell populations and proviral states are relevant to rebound, chronic inflammation, and neurological injury.
- Determine the contribution of defective but transcriptionally active proviruses to glial activation or neuronal dysfunction.
- Develop CNS-friendly delivery systems which retain cell specificity and a strong safety margin.
- Incorporate neuronal, glial, and neuroimmune endpoints into reservoir-targeting studies from the outset.
- Test combination strategies designed to reduce viral persistence while preserving or restoring CNS health.

## Conclusions

In summary, a cure for HIV must ultimately work across tissues, cell types, and biological timescales. In the CNS, this means that successful interventions may need to do more than eliminate replication-competent virus. They may also need to silence harmful viral transcription, restore a healthier neuroimmune environment, and protect neural systems that remain vulnerable despite systemic viral suppression.

## Key References

- Dave RS, Oludipe AA, Pasipanodya EC, Sherman S, Wilson SH, Gabuzda DH, Churchill MJ, Angelovich TA, Gorelick RJ, Gianella S, et al. The brain as an HIV reservoir: Recent findings using autopsy tissues from people with HIV. PLOS Pathogens 2026; 22:e1014446.
  - ○ This comprehensive review highlights key findings from post-mortem studies defining HIV reservoir size across the NNTC and Last Gift cohorts.
- Liu H, Liao S, Chen C, Huo W, Safaei Z, Rocchi A, Huang Y, Sariyer IK, Collins ME, Lawrence JM, et al. In Vivo Genome Editing of Central Nervous System SIV Reservoirs in ART-Suppressed Rhesus Macaques. Molecular Therapy Nucleic Acids 2026:103063.
  - ○ This recent study demonstrates the efficacy of CRISPR-based editing of intact CNS reservoirs in ART-suppressed SIV-infected rhesus macaques.
- Bohannon DG, Zablocki-Thomas LD, Leung ES, Dupont JK, Hattler JB, Kowalewska J, Zhao M, Luo J, Salemi M, Amedee AM, et al. CSF1R inhibition depletes brain macrophages and reduces brain virus burden in SIV-infected macaques. Brain 2024; 147:3059-69.
  - ○ Therapeutic inhibition of CSF1R with BLZ945 was demonstrated to reduce levels of SIV DNA and number of perivascular macrophages in the brain of SIV-infected animals.
- Li X, Li D, Tang Y, Nearing M, Varco-Merth B, Chen H, Smith D, Gianella S, Archin NM, Okoye AA, et al. Histone decrotonylation plays a distinct role in HIV latency. Science Advances 2026; 12:eaec0149.
  - ○ HIV transcription was upregulated in latently infected microglia and CD4+ T cells from PWH when treated with the selective histone decrotonylation inhibitor citarinostat.

## Data Availability

No datasets were generated or analysed during the current study.

## Declarations

### AI Usage

Generative AI was not used in the preparation of the manuscript.
